# The role of statins in the treatment of lung cancer with epidermal growth factor receptor-tyrosine kinase inhibitors: a protocol for systematic review and meta-analysis

**DOI:** 10.3389/fmed.2026.1792449

**Published:** 2026-06-18

**Authors:** Surui Yuan, Bingxue Li, Yi Jiang, Liping Shen, Zhenyue Fu, Lingshuang Liu

**Affiliations:** Longhua Hospital, Shanghai University of Traditional Chinese Medicine, Shanghai, China

**Keywords:** epidermal growth factor receptor-tyrosine kinase inhibitor, lung cancer, meta-analysis, statin, systematic review

## Abstract

**Introduction:**

Epidermal growth factor receptor-tyrosine kinase inhibitors (EGFR-TKIs) have substantially improved outcomes in patients with EGFR-mutant non-small cell lung cancer (NSCLC). However, both primary and acquired resistance limit their long-term efficacy. Statins have shown potential to modulate cholesterol metabolism and EGFR-related signaling, and may therefore influence treatment outcomes in this population. Current clinical evidence remains inconsistent and heterogeneous. This study aims to systematically evaluate whether statin use is associated with improved efficacy outcomes in patients with EGFR-mutant NSCLC receiving EGFR-TKIs, while also assessing reported adverse events as a supplementary safety outcome.

**Methods:**

This review will follow the Preferred Reporting Items for Systematic Review and Meta-Analysis Protocols guidelines. PubMed, EMBASE, Cochrane Library, Web of Science, China National Knowledge Infrastructure, China Science and Technology Journal Database, Wanfang Data, and the Chinese Biomedical Literature Database will be searched for randomized controlled trials and observational studies evaluating statin exposure in patients with EGFR-mutant NSCLC receiving EGFR-TKIs. The websites of key academic societies and clinical trial registries will also be reviewed. Two reviewers will independently select studies and extract data. Risk of bias will be assessed using the Cochrane Risk-of-Bias tool 2.0 and Risk of Bias in Non-randomized Studies of Interventions (ROBINS-I), with particular attention to confounding and time-related biases in observational studies. Overall survival will be the primary outcome, and progression-free survival will be the key secondary outcome. Randomized and observational studies will be synthesized separately, and subgroup, sensitivity, and reporting bias analyses will be conducted where appropriate.

**Discussion:**

Preclinical studies suggest that statins may enhance the antitumor activity of EGFR-TKIs, but the clinical evidence remains inconsistent. This protocol will synthesize available evidence to evaluate whether statin exposure is associated with the efficacy of EGFR-TKIs in EGFR-mutant NSCLC, and to explore potential sources of heterogeneity across studies.

**Systematic review registration:**

https://www.crd.york.ac.uk/PROSPERO/view/CRD420251111062, PROSPERO CRD420251111062.

## 1 Introduction

### 1.1 Description of the condition

According to global cancer statistics, lung cancer ranks first in both incidence and mortality among all malignancies, with approximately 2.5 million new cases and 1.8 million deaths annually, accounting for 12.4 and 18.7% of the global totals, respectively (1). Non-small cell lung cancer (NSCLC) makes up most lung cancer cases (2). As 40–60% Asian patients and 10–15% Western patients (3) demonstrate with epidermal growth factor receptor (EGFR) mutations, which lead to changes in the intracellular region of EGFR and cause its excessive activation to promote cancer progression, EGFR-tyrosine kinase inhibitors (EGFR-TKIs) are frequently used for lung cancer treatment, especially as adjuvant therapy following surgical resection of Ib-IIIB NSCLC patients or systemic therapy for advanced or metastatic disease. The use of EGFR-TKIs, with fewer side effects than chemotherapy, has greatly improved outcomes for NSCLC patients with EGFR mutations (4–7). However, 20–30% of patients with EGFR mutations do not respond from the start (primary resistance) (8, 9), while others stop responding after 10–18 months (acquired resistance) (5, 10). Primary resistance to EGFR-TKIs often occurs in patients with wild-type EGFR or certain mutations (such as exon 20 insertions), or due to activation of bypass pathways (such as KRAS or PIK3CA mutations, or PTEN loss). Acquired resistance usually results from secondary mutations in EGFR (most notably T790M or C797S) or alternative pathway activation (such as MET or HER2 amplification). These mechanisms keep the downstream RAS/MEK/ERK and PI3K/Akt pathways constantly active, thereby leading to drug resistance (11–13). The treatment for EGFR-TKI resistance is related to the specific mechanism and has limited efficacy (14). New strategies must be developed to enhance the performance of EGFR-TKIs, thereby helping patients live longer and better.

Metabolism changes are attracting more and more attention in cancer treatment, including for drug resistance nowadays. It has been reported that reprogramming of cholesterol metabolism is a key metabolic feature of EGFR-TKI resistance in NSCLC, regardless of primary resistance or secondary resistance. EGFR-TKI-induced resistant cells exhibit significantly higher cholesterol levels than sensitive cells (15). Cholesterol levels in lipid rafts (LRs) are also significantly higher in gefitinib-resistant NSCLC cell lines than in gefitinib-sensitive lines (16). Cholesterol can inhibit the binding of gefitinib to EGFR, block EGFR signaling transduction through stabilizing LRs, or activate alternative pathways, which can all lead to drug resistance (16–19). Depletion of cholesterol can restore cellular sensitivity to gefitinib (16, 19). So cholesterol-directed therapy may represent a potential avenue for further investigation in EGFR-TKI resistance (17).

### 1.2 Description of the intervention and how it might work

Among all the existing approved cholesterol-lowering drugs, statins are the most frequently used ones (20, 21). Statins are inhibitors of 3-hydroxy-3-methylglutaryl-coenzyme A (HMG-CoA) reductase, which work by reducing the conversion of HMG-CoA to mevalonate and thus inhibit the production of cholesterol. Statins can be further categorized into hydrophilic, lipophilic, and amphiphilic types, a property that influences their tissue distribution and thus presents with different drug–drug interactions and toxicities (22). There are studies focusing on statins’ effect on EGFR-TKI resistance. Statins can prenylate important proteins like Ras and Rho, suppress their activation and downstream signal transduction so as to inhibit EGFR signaling (15, 23–33). Combined statin and EGFR-TKI treatment disrupts AKT-mediated SREBP-1 signaling, yielding synergistic antitumor effects in both cellular and animal models (34). These findings provide a rationale for investigating statin exposure in patients receiving EGFR-TKIs (35). Several clinical studies have evaluated statin exposure in patients treated with EGFR-TKIs (35–38), but the reported associations with survival outcomes remain inconsistent (39–42). Differences in EGFR mutation subtype, treatment line, statin class, exposure timing, and study design may contribute to the observed variability.

### 1.3 Why is it important to do this review

Although a previous meta-analysis published in 2019 suggested that statins might improve outcomes in lung cancer patients receiving TKI therapy, the evidence specifically relevant to EGFR-mutant NSCLC treated with EGFR-TKIs remained limited (43). The TKI-related subgroup included only a small number of studies, substantial heterogeneity was present in key outcomes, and stratified analyses by EGFR mutation subtype, treatment line, and statin exposure pattern were insufficient. In addition, several clinically relevant studies have been published in recent years, warranting an updated and more focused evidence synthesis. Therefore, this protocol aims to systematically evaluate whether statin use is associated with improved clinical outcomes in patients with EGFR-mutant NSCLC receiving EGFR-TKIs. Overall survival is the primary outcome, and progression-free survival is the key secondary endpoint. Other efficacy outcomes will be considered exploratory, while reported adverse events will be assessed as a supplementary safety outcome.

## 2 Method

### 2.1 Protocol registration

This systematic review protocol will follow the Preferred Reporting Items for Systematic Review and Meta-Analysis Protocols (PRISMA-P) 2015 extension (44, 45) and is available in Supplementary file. The review protocol was registered with PROSPERO (CRD420251111062). The final report will follow the PRISMA 2020 statement (46).

### 2.2 Search methods

The following databases will be systematically searched: PubMed, EMBASE, the Cochrane Library, Web of Science, China National Knowledge Infrastructure (CNKI), China Science and Technology Journal Database (VIP), Wanfang Data Knowledge Service Platform, and the Chinese Biomedical Literature Database (CBM). In addition, the official websites of key academic societies will be reviewed, including those of the European Society for Medical Oncology (ESMO), the American Society of Clinical Oncology (ASCO), the American Association for Cancer Research (AACR), and the Chinese Society of Clinical Oncology (CSCO). Clinical trial registries will be searched. Reference lists of included articles and relevant reviews will be manually screened. No language or publication date restrictions will be applied. The search will use medical subject headings (MeSH) combined with free-text terms. Key terms include “non-small cell lung cancer,” “EGFR-TKI,” “epidermal growth factor receptor tyrosine kinase inhibitor,” “statin,” and “HMG-CoA reductase inhibitor.” In addition to generic statin-related terms, the search strategy will include individual statin names such as atorvastatin, simvastatin, rosuvastatin, pravastatin, fluvastatin, lovastatin, and pitavastatin to improve retrieval sensitivity. Boolean operators (AND, OR, NOT) will be used to refine searches. The complete search strategy for PubMed is provided in Supplementary file.

### 2.3 Study inclusion criteria

Inclusion criteria are defined using the PICOS (Participants, Interventions, Comparisons, Outcomes, Study design) framework.

#### 2.3.1 Types of participants

Patients with histologically or cytologically confirmed EGFR-mutant NSCLC receiving EGFR-TKI therapy will be included. Studies enrolling mixed NSCLC populations will be eligible only if data for the EGFR-mutant EGFR-TKI-treated subgroup can be extracted separately. Clinical stage, pathological subtype, treatment setting, and line of therapy will be recorded for subgroup or sensitivity analyses where appropriate.

#### 2.3.2 Types of interventions

The intervention of interest is statin exposure in patients receiving EGFR-TKI therapy. Data on statin type, dose, timing of initiation, duration of use, and overlap with EGFR-TKI treatment will be extracted whenever available. Because exposure definitions may vary across studies, analyses will take these differences into account where data permit.

#### 2.3.3 Types of comparison groups

In randomized trials, the comparison group may include placebo or non-statin control arms. In observational studies, the comparison group will consist of patients without statin exposure during the defined observation window. These comparator definitions will be handled separately according to study design.

#### 2.3.4 Types of outcome measures

The primary outcome is overall survival (OS), defined as the time from treatment initiation or the study-defined index date to death from any cause. The key secondary outcome is progression-free survival (PFS), defined as the time from treatment initiation or the study-defined index date to disease progression or death from any cause. Exploratory efficacy outcomes include cancer-specific survival (CSS), objective response rate (ORR), disease control rate (DCR), and, where relevant to curative treatment settings, disease-free survival (DFS) and recurrence-free survival (RFS). Reported adverse events (AEs) (47) will be assessed as a supplementary safety outcome. Included studies must report at least one of these outcomes together with clear definitions and sufficient statistical data for extraction.

#### 2.3.5 Types of studies

Randomized controlled trials (RCTs) and observational studies, such as cohort and case–control studies, will be included in this study.

### 2.4 Exclusion criteria

Studies will be excluded for the following reasons: (1) the study population is not clearly relevant to EGFR-mutant NSCLC treated with EGFR-TKIs; (2) subgroup data for the target population cannot be extracted; (3) statin exposure is inadequately defined or not distinguishable; (4) key outcome data are unavailable after reasonable attempts to contact the authors; (5) duplicate publications or overlapping cohorts, in which case the most complete and informative report will be retained; and (6) non-original research, including reviews, editorials, commentaries, and conference abstracts without sufficient data for extraction.

### 2.5 Data collection and analysis

#### 2.5.1 Study selection

**Figure 1:**
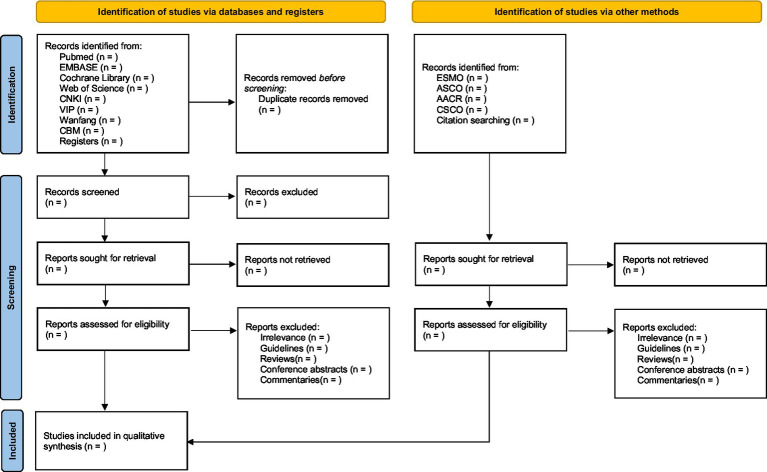
PRISMA flow diagram of study selection for the meta-analysis.

Search results will be exported to EndNote 21 for duplicate removal. Two reviewers will independently screen titles and abstracts based on the inclusion criteria. Studies identified as potentially eligible will undergo full-text review. Disagreements between reviewers will be resolved through discussion. If consensus is not reached, a third reviewer will be consulted. The number of records from each source and the reasons for exclusion will be recorded. The study selection process will be summarized in a PRISMA flow diagram (Figure 1) (46).

#### 2.5.2 Data extraction and management

A standardized data extraction form will be developed to collect the following information: (1) study characteristics: author, publication year, study design, country, eligibility criteria, sample size, and follow-up time; (2) participant characteristics: age, sex, race, smoking status, Eastern Cooperative Oncology Group Performance Status (ECOG PS), pathological subtype, clinical stage, EGFR mutation subtype, treatment history, comorbidities, and line of therapy; (3) intervention-related characteristics: statin type, dose, timing of initiation, duration of exposure, overlap duration with EGFR-TKI treatment, type of EGFR-TKI, and adverse events (48, 49); (4) key items required for risk of bias assessment, including adjustment for major confounders and handling of time-related bias; and (5) effect estimates for outcome measures, including adjusted and unadjusted hazard ratios, risk ratios, *p*-values, and 95% confidence intervals (CIs), where available. Data will be extracted independently by two reviewers. Disagreements will be resolved through discussion or by consultation with a third reviewer to ensure consistency.

#### 2.5.3 Risk of bias assessment

The Cochrane Risk-of-Bias tool 2.0 (RoB 2) will be used to assess the methodological quality of included RCTs (50, 51). RoB 2 examines five domains of bias: the randomization process, deviations from intended interventions, missing outcome data, measurement of the outcome, and selection of the reported result. Each domain is rated as “low risk,” “some concerns,” or “high risk.”

For observational studies, the Risk of Bias in Non-randomized Studies of Interventions (ROBINS-I) tool will be applied (52). It covers seven bias domains: confounding, selection of participants, classification of interventions, deviations from intended interventions, missing data, measurement of outcomes, and selection of the reported result. Judgments are classified as “low risk,” “moderate risk,” “serious risk,” “critical risk,” or “no information.” Two reviewers will independently carry out the risk of bias evaluation. Any disagreements will be resolved through discussion or by consulting a third reviewer. For observational studies, special attention will be given to confounding by indication, cardiovascular comorbidity, baseline performance status, concomitant medications, healthcare utilization, healthy-user effects, and time-related biases such as immortal time bias.

#### 2.5.4 Measures of treatment effect

For time-to-event outcomes (including OS, PFS, and CSS), hazard ratios (HRs) with 95% CIs will be used. HR values will be extracted directly from publications when available. If not reported, Engauge Digitizer will be used to extract survival rates at various time points from Kaplan–Meier curves, and HRs with standard errors (SEs) will be calculated using the spreadsheet developed by Jayne F. Tierney et al. (53, 54). For dichotomous outcomes (such as ORR, DCR, and AEs), risk ratios (RRs) with 95% CIs will be applied. For observational studies, adjusted effect estimates will be preferentially extracted over crude estimates whenever available.

#### 2.5.5 Handling of missing data

For missing or unclear data, authors will be contacted by email to obtain the data. If the required data remain unavailable after contact, studies with missing data will be excluded from analysis. A sensitivity analysis will then be conducted to examine whether the missing data affect the robustness of the meta-analysis results.

#### 2.5.6 Assessment of heterogeneity

Heterogeneity will be assessed from clinical, methodological, and statistical perspectives. Forest plots generated in Review Manager 5.4 will be used to visually examine the overlap of confidence intervals across studies. Cochran’s Q test and the *I*^2^ statistic will be applied for statistical evaluation (55). A *p*-value < 0.10 in Cochran’s *Q* test will indicate the presence of heterogeneity. The *I*^2^ statistic will be used to quantify the proportion of total variation across studies that is due to heterogeneity rather than chance. *I*^2^ values of 0–25%, 25–50%, 50–75%, and 75–100% will be interpreted as low, moderate, substantial, and considerable heterogeneity, respectively. However, decisions regarding quantitative pooling will not rely solely on statistical heterogeneity. Differences in study design, patient population, EGFR mutation subtype, treatment line, statin exposure definition, comparator structure, and outcome ascertainment will also be considered when determining whether studies are sufficiently comparable for meta-analysis.

#### 2.5.7 Data synthesis strategy

All analyses will be conducted using Review Manager 5.4. Randomized trials and observational studies will be synthesized separately. Quantitative pooling will only be performed when studies are judged sufficiently comparable in terms of population, intervention, comparator, and outcome definitions. Where substantial clinical or methodological heterogeneity precludes meaningful pooling, a narrative synthesis will be provided instead. When meta-analysis is appropriate, a random-effects model will generally be preferred when clinical heterogeneity is expected, even if statistical heterogeneity is not extreme. Studies with poorly defined exposure timing or inadequate control of time-related bias will be interpreted with particular caution. Forest plots will be generated to visually present the results.

#### 2.5.8 Assessment of reporting bias

If ten or more studies are included, funnel plots will be used to assess symmetry in effect size distribution. Egger’s test will be applied to examine publication bias, with a *p*-value < 0.05 indicating its presence. If identified, the trim-and-fill method will be used to evaluate the potential impact of missing studies on pooled effect estimates (56).

#### 2.5.9 Subgroup analysis

Prespecified subgroup analyses will be conducted, where sufficient data are available, to explore clinically relevant sources of heterogeneity. These subgroups include EGFR mutation subtype, line of EGFR-TKI therapy, type of EGFR-TKI, statin class, statin exposure timing and overlap duration, disease stage, and study design. These analyses will be interpreted cautiously, particularly when the number of available studies is limited.

#### 2.5.10 Sensitivity analysis

Sensitivity analysis will be performed using the leave-one-out method to evaluate the influence of individual studies on the overall effect size. If the exclusion of a particular study leads to substantially different pooled effect estimates or contradictory conclusions, the result will be considered unstable and of low robustness.

#### 2.5.11 Certainty of evidence

The quality of evidence will be assessed using the Grading of Recommendations Assessment, Development and Evaluation (GRADE) system. This approach considers five factors that can lower the evidence quality—risk of bias, inconsistency, indirectness, imprecision, and publication bias—and three factors that can raise it—large magnitude of effect, dose–response gradient, and plausible confounding. Based on this evaluation, the overall certainty will be categorized into four levels: high, moderate, low, or very low (57, 58).

#### 2.5.12 Ethics and dissemination

As this study is a secondary analysis of published data and does not involve direct patient recruitment, ethical approval is not required. The results will be published in a peer-reviewed journal.

## 3 Discussion

The antitumor potential of statins has attracted considerable interest, and some clinical studies have reported favorable associations between statin use and outcomes in NSCLC (43, 59–62). However, whether statin exposure is associated with improved prognosis specifically in patients with EGFR-mutant NSCLC receiving EGFR-TKIs remains uncertain. Published findings are inconsistent, and the observed differences may reflect variations in mutation subtype, treatment context, exposure definition, and residual confounding. This protocol is designed to address these uncertainties through a more focused and methodologically explicit evidence synthesis.

### 3.1 Strengths of our study

This protocol has several strengths. First, it focuses specifically on patients with EGFR-mutant NSCLC receiving EGFR-TKIs, thereby narrowing the clinical question and improving interpretability. Second, prespecified subgroup analyses will be conducted, where sufficient data are available, according to clinically relevant factors such as EGFR mutation subtype, line of therapy, EGFR-TKI type, statin class, and exposure timing. Third, the protocol distinguishes randomized and observational evidence and explicitly addresses key sources of bias and heterogeneity, which may improve the rigor of the planned synthesis.

### 3.2 Future directions

Although preclinical findings suggest that statins may influence EGFR-related resistance pathways, their clinical role in EGFR-mutant NSCLC treated with EGFR-TKIs remains uncertain. The present protocol is intended to provide a more focused and methodologically rigorous synthesis of the available evidence and to clarify whether any observed associations differ across clinically relevant subgroups.

Future studies should move beyond broad retrospective comparisons and prioritize biomarker-informed prospective research in clearly defined populations. In particular, further studies should examine whether the association between statin exposure and outcomes differs according to EGFR mutation subtype, such as exon 19 deletion versus L858R, EGFR-TKI generation, line of therapy, and the timing and duration of statin use. Prospective studies should also incorporate rigorous control of cardiovascular comorbidities, concomitant medications, healthy-user effects, and time-related biases. Such work will be necessary to determine whether statins have a clinically meaningful adjunctive role in EGFR-mutant NSCLC treated with EGFR-TKIs.

## Glossary

AACR: American Association for Cancer Research
AE: adverse event
ASCO: American Society of Clinical Oncology
CBM: Chinese biomedical literature database
CI: confidence interval
CNKI: china national knowledge infrastructure
CSCO: Chinese Society of Clinical Oncology
CSS: cancer-specific survival
CTCAE: common terminology criteria for adverse events
DCR: disease control rate
DFS: disease-free survival
ECOG PS: eastern cooperative oncology group performance status
EGFR-TKI: epidermal growth factor receptor-tyrosine kinase inhibitor
ESMO: European Society for Medical Oncology
GRADE: grading of recommendations assessment, development, and evaluation
HMG-CoA: 3-hydroxy-3-methylglutaryl-coenzyme A
HR: hazard ratio
LR: lipid raft
MeSH: medical subject headings
NSCLC: non-small cell lung cancer
ORR: objective response rate
OS: overall survival
PFS: progression-free survival
PICOS: participants, interventions, comparisons, outcomes, study design
PRISMA-P: preferred reporting items for systematic review and meta-analysis protocols
PRO: patient-reported outcome
RCT: randomized controlled trial
RFS: recurrence-free survival
RoB 2: Cochrane risk-of-bias tool 2.0
ROBINS-I: Risk of bias in non-randomized studies-of interventions
RR: risk ratio
SE: standard error
VIP: China science and technology journal database
