# Supplementary material for: The role of statins in the treatment of lung cancer with epidermal growth factor receptor-tyrosine kinase inhibitors: a protocol for systematic review and meta-analysis
Source: Front Med (Lausanne). 2026 Jun 18;13:1792449. doi: 10.3389/fmed.2026.1792449 (PMC13323136; doi:10.3389/fmed.2026.1792449)
Supplement: Supplementary file 1 [file Supplementary_file_1.docx]

**PUBMED search strategy**

| #1 | "Carcinoma, Non-Small-Cell Lung"[Mesh] |
| --- | --- |
| #2 | (Carcinoma, Non Small Cell Lung[Title/Abstract] OR Carcinomas, Non-Small-Cell Lung[Title/Abstract] OR Lung Carcinoma, Non-Small-Cell[Title/Abstract] OR Lung Carcinomas, Non-Small-Cell[Title/Abstract] OR Non-Small-Cell Lung Carcinomas[Title/Abstract] OR Carcinoma, Non-Small Cell Lung[Title/Abstract] OR Non-Small Cell Lung Cancer[Title/Abstract] OR Non-Small-Cell Lung Carcinoma[Title/Abstract] OR Non Small Cell Lung Carcinoma[Title/Abstract] OR Nonsmall Cell Lung Cancer[Title/Abstract] OR Non-Small Cell Lung Carcinoma[Title/Abstract]) |
| #3 | #1OR#2 |
| #4 | "Hydroxymethylglutaryl-CoA Reductase Inhibitors"[Mesh] |
| #5 | (Hydroxymethylglutaryl CoA Reductase Inhibitors[Title/Abstract] OR Inhibitors, Hydroxymethylglutaryl-CoA Reductase[Title/Abstract] OR Reductase Inhibitors, Hydroxymethylglutaryl-CoA[Title/Abstract] OR HMG-CoA Reductase Inhibitor[Title/Abstract] OR HMG CoA Reductase Inhibitor[Title/Abstract] OR Statin[Title/Abstract] OR Statins[Title/Abstract] OR Inhibitors, HMG-CoA Reductase[Title/Abstract] OR Inhibitors, HMG CoA Reductase[Title/Abstract] OR Reductase Inhibitors, HMG-CoA[Title/Abstract] OR HMG-CoA Reductase Inhibitors[Title/Abstract] OR HMG CoA Reductase Inhibitors[Title/Abstract] OR Inhibitors, Hydroxymethylglutaryl-Coenzyme A[Title/Abstract] OR Hydroxymethylglutaryl-Coenzyme A Inhibitors[Title/Abstract] OR Inhibitors, Hydroxymethylglutaryl Coenzyme A[Title/Abstract] OR Inhibitors, Hydroxymethylglutaryl-CoA[Title/Abstract] OR Hydroxymethylglutaryl-CoA Inhibitors[Title/Abstract] OR Inhibitors, Hydroxymethylglutaryl CoA[Title/Abstract] OR Hydroxymethylglutaryl-CoA Reductase Inhibitor[Title/Abstract] OR Hydroxymethylglutaryl CoA Reductase Inhibitor[Title/Abstract] OR Reductase Inhibitor, Hydroxymethylglutaryl-CoA[Title/Abstract] OR Statins, HMG-CoA[Title/Abstract] OR HMG-CoA Statins[Title/Abstract] OR Statins, HMG CoA[Title/Abstract]) |
| #6 | #4OR#5 |
| #7 | (targeted therapy[Title/Abstract] OR epidermal growth factor receptor-tyrosine kinase inhibitor[Title/Abstract] OR EGFR-TKI[Title/Abstract] OR Gefitinib[Title/Abstract] OR Erlotinib[Title/Abstract] OR Icotinib[Title/Abstract] OR Afatinib[Title/Abstract] OR Dacomitinib[Title/Abstract] OR Osimertinib[Title/Abstract] OR Almonertinib[Title/Abstract] OR Furmonertinib[Title/Abstract] OR Abivertinib[Title/Abstract] OR Lazertinib[Title/Abstract] OR Olmutinib[Title/Abstract] OR Befotertinib[Title/Abstract] OR Limertinib[Title/Abstract] OR Rezivertinib[Title/Abstract] OR Rilertinib[Title/Abstract] OR zorifertinib[Title/Abstract]) |
| #8 | #3AND#6AND#7 |
| #9 | (animals[MeSH]) NOT (humans[MeSH]) |
| #10 | #8NOT#9 |
